# Bis(2-amino-5-bromo­pyridinium) fumarate dihydrate

**DOI:** 10.1107/S1600536810030989

**Published:** 2010-08-11

**Authors:** Ching Kheng Quah, Madhukar Hemamalini, Hoong-Kun Fun

**Affiliations:** a X-ray Crystallography Unit, School of Physics, Universiti Sains Malaysia, 11800 USM, Penang, Malaysia

## Abstract

In the title compound, 2C_5_H_6_BrN_2_^+^·C_4_H_2_O_4_^2−^·2H_2_O, the complete fumarate dianion is generated by crystallographic inversion symmetry. The cation is approximately planar, with a maximum deviation of 0.036 (1) Å. In the anion, the carboxyl­ate group is twisted slightly away from the attached plane; the dihedral angle between carboxyl­ate and (*E*)-but-2-ene planes is 6.11 (14)°. In the crystal, the carboxyl­ate O atoms form bifurcated (N—H⋯O and C—H⋯O) and N—H⋯O hydrogen bonds with the cations. The crystal packing is stabilized by *R*_2_^2^(8) ring motifs which are generated by pairs of N—H⋯O hydrogen bonds. The crystal structure is further consolidated by water mol­ecules *via* O(water)—H⋯O and N—H⋯O(water) hydrogen bonds. The components are linked by these inter­actions into three-dimensional network.

## Related literature

For details of hydrogen bonding, see: Goswami & Ghosh (1997 ▶); Goswami *et al.* (1998 ▶). For applications of fumaric acid, see: Batchelor *et al.* (2000 ▶). For related structures, see: Büyükgüngör *et al.* (2004 ▶); Büyükgüngör & Odabąsoğlu (20065); Hemamalini & Fun, (2010**a*,b*); Quah *et al.* (2008 ▶; 2010**a*,b*). For bond-length data, see: Allen *et al.* (1987 ▶). For the stability of the temperature controller used in the data collection, see: Cosier & Glazer (1986 ▶). For hydrogen-bond motifs, see: Bernstein *et al.* (1995 ▶).
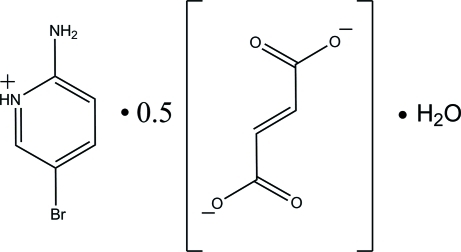


## Experimental

### 

#### Crystal data

2C_5_H_6_BrN_2_^+^·C_4_H_2_O_4_^2−^·H_2_O
*M* *_r_* = 498.14
Monoclinic, 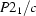

*a* = 8.3717 (1) Å
*b* = 16.5354 (2) Å
*c* = 6.7846 (1) Å
β = 108.336 (1)°
*V* = 891.50 (2) Å^3^
*Z* = 2
Mo *K*α radiation
μ = 4.59 mm^−1^
*T* = 100 K
0.39 × 0.15 × 0.12 mm

#### Data collection

Bruker SMART APEXII CCD area-detector diffractometer
Absorption correction: multi-scan (*SADABS*; Bruker, 2009 ▶) *T*_min_ = 0.271, *T*_max_ = 0.618
15040 measured reflections
3942 independent reflections
3252 reflections with *I* > 2σ(*I*)
*R*_int_ = 0.028

#### Refinement

*R*[*F* ^2^ > 2σ(*F* ^2^)] = 0.026
*wR*(*F* ^2^) = 0.059
*S* = 1.03
3942 reflections
138 parameters
H atoms treated by a mixture of independent and constrained refinement
Δρ_max_ = 0.54 e Å^−3^
Δρ_min_ = −0.39 e Å^−3^

### 

Data collection: *APEX2* (Bruker, 2009 ▶); cell refinement: *SAINT* (Bruker, 2009 ▶); data reduction: *SAINT*; program(s) used to solve structure: *SHELXTL* (Sheldrick, 2008 ▶); program(s) used to refine structure: *SHELXTL*; molecular graphics: *SHELXTL*; software used to prepare material for publication: *SHELXTL* and *PLATON* (Spek, 2009 ▶).

## Supplementary Material

Crystal structure: contains datablocks global, I. DOI: 10.1107/S1600536810030989/bt5312sup1.cif

Structure factors: contains datablocks I. DOI: 10.1107/S1600536810030989/bt5312Isup2.hkl

Additional supplementary materials:  crystallographic information; 3D view; checkCIF report

## Figures and Tables

**Table 1:** Hydrogen-bond geometry (Å, °)

*D*—H⋯*A*	*D*—H	H⋯*A*	*D*⋯*A*	*D*—H⋯*A*
N1—H1*N*1⋯O1	0.902 (18)	1.815 (18)	2.7136 (14)	174.1 (19)
N2—H2*N*2⋯O1*W*^i^	0.82 (2)	2.11 (2)	2.9143 (16)	169.7 (19)
N2—H1*N*2⋯O2	0.893 (19)	1.946 (19)	2.8348 (15)	173.2 (19)
O1*W*—H2*W*1⋯O1^ii^	0.77 (2)	2.07 (2)	2.8213 (15)	169 (2)
O1*W*—H1*W*1⋯O1^iii^	0.82 (3)	1.99 (3)	2.7865 (14)	167 (3)
C3—H3*A*⋯O2^iv^	0.93	2.41	3.3089 (17)	162

Symmetry codes: (i) 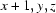; (ii) 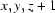; (iii) 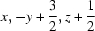; (iv) 
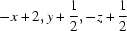
.

**Fig. 1.**
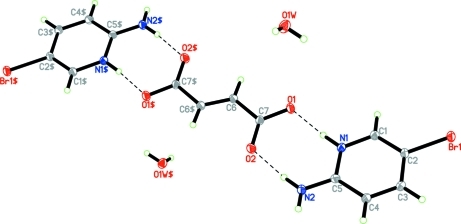
The molecular structure of the title compound showing 50% probability displacement ellipsoids for non-H atoms and the atom-numbering scheme. Symmetry code: ($) -x + 1, -y + 1, -z. Intramolecular interactions are shown as dashed lines.

**Fig. 2.**
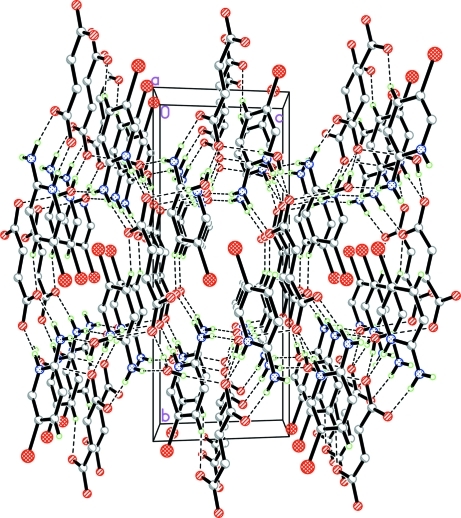
The crystal structure of the title compound viewed along the a axis. H atoms not involved in intermolecular interactions (dashed lines) have been omitted for clarity.

## supplementary crystallographic information

### Comment

Hydrogen bonding plays a key role in molecular recognition (Goswami & Ghosh, 1997) and crystal engineering research (Goswami *et al.*, 1998). Fumaric acid, the *E* isomer of butenedioic acid, is of interest since it is known to form supramolecular assemblies with *N*-aromatic compounds (Batchelor *et al.*, 2000). It tends to form infinite chains arranged in a nearly coplanar manner *via* pairs of strong O—H···O hydrogen bonds. The crystal structures of 2-aminopyridinium-fumarate-fumaric acid (2/1/1) (Büyükgüngör *et al.*, 2004) and 2,6-diamino pyridinium hydrogen fumarate (Büyükgüngör & Odabąsoǧlu, 2006) have been reported in the literature. We have recently reported the crystal structures of 2-amino-5-chloropyridine-fumaric acid (1/2) (Hemamalini & Fun, 2010*a*) and 2-amino-4-methylpyridinium (*E*)-3-carboxyprop-2-enoate (Hemamalini & Fun, 2010*b*) from our laboratory. In order to study some interesting hydrogen bonding interactions, the synthesis and structure of the title compound, (I), is presented here.

The asymmetric unit of title compound (Fig. 1), consist of a protonated 2-amino-5-bromopyridinium cation, a half molecule of fumarate anion and a water molecule. The fumarate anion is lying about an inversion center (symmetry code: -*x* + 1, -*y* + 1, -*z*). In the 2-amino-5-bromopyridinium cation, protonatation of N1 atom has lead to a slight increase in the C1–N1–C5 angle to 122.64 (11)°. The 2-amino-5-bromopyridinium cation is approximately planar, with a maximum deviation of 0.036 (1) Å for atom N2. In fumarate anion, C6/C7/C6A/C7A plane is planar with an r.m.s deviation of <0.001 (1) Å. This plane makes a dihedral angle of 6.90 (6)° with 2-amino-5-bromopyridinium cation. In the anion, the carboxylate group is twisted slightly away from the attached plane; the dihedral angle between C6/C7/C6A/C7A and O1/O2/C6/C7 planes is 6.11 (14)°.

In the crystal packing (Fig. 2), the carboxylate oxygen atoms, O2 and O3 form bifurcated (N2–H1N2···O2 and C3–H3A···O2) and N1–H1N1···O1 hydrogen bonds, respectively, with cations. The crystal packing is stabilized by *R*_2_^2^(8) ring motifs (Bernstein *et al.*, 1995) which are generated by pairs of N–H···O hydrogen bonds (Table 1). The crystal structure is further consolidated by water molecules *via* O1W–H1W1···O1, O1W–H2W1···O1 and N2–H2N2···O1W hydrogen bonds. The anions, cations and water molecules are linked by these interactions into three-dimensional network.

### Experimental

A hot methanol solution (20 ml) of 2-amino-5-bromopyridine (86 mg, Aldrich) and fumaric acid (58 mg, Merck) was mixed and warmed over a magnetic stirrer hotplate for a few minutes. The resulting solution was allowed to cool slowly at room temperature and crystals of the title compound appeared after a few days.

### Refinement

O– and N– bound H atoms were located in a difference Fourier map and allowed to refined freely. The rest of the hydrogen atoms were positioned geometrically and refined using a riding model with C—H = 0.93 Å and *U*_iso_(H) = 1.2 *U*_eq_(C). The highest residual electron density peak is located at 0.66 Å from C6 and the deepest hole is located at 1.19 Å from Br1.

### Crystal data

**Table:**

2C_5_H_6_BrN_2_^+^·C_4_H_2_O_4_^2^^−^·2H_2_O	*F*(000) = 496
*M**_r_* = 498.14	*D*_x_ = 1.856 Mg m^−^^3^
Monoclinic, *P*2_1_/*c*	Mo *K*α radiation, λ = 0.71073 Å
Hall symbol: -P 2ybc	Cell parameters from 5779 reflections
*a* = 8.3717 (1) Å	θ = 2.6–34.6°
*b* = 16.5354 (2) Å	µ = 4.59 mm^−^^1^
*c* = 6.7846 (1) Å	*T* = 100 K
β = 108.336 (1)°	Block, colourless
*V* = 891.50 (2) Å^3^	0.39 × 0.15 × 0.12 mm
*Z* = 2	

### Data collection

**Table:**

Bruker SMART APEXII CCD area-detector diffractometer	3942 independent reflections
Radiation source: fine-focus sealed tube	3252 reflections with *I* > 2σ(*I*)
graphite	*R*_int_ = 0.028
φ and ω scans	θ_max_ = 35.2°, θ_min_ = 2.5°
Absorption correction: multi-scan (*SADABS*; Bruker, 2009)	*h* = −13→12
*T*_min_ = 0.271, *T*_max_ = 0.618	*k* = −26→23
15040 measured reflections	*l* = −10→10

### Refinement

**Table:**

Refinement on *F*^2^	Primary atom site location: structure-invariant direct methods
Least-squares matrix: full	Secondary atom site location: difference Fourier map
*R*[*F*^2^ > 2σ(*F*^2^)] = 0.026	Hydrogen site location: inferred from neighbouring sites
*wR*(*F*^2^) = 0.059	H atoms treated by a mixture of independent and constrained refinement
*S* = 1.03	*w* = 1/[σ^2^(*F*_o_^2^) + (0.0265*P*)^2^ + 0.2048*P*] where *P* = (*F*_o_^2^ + 2*F*_c_^2^)/3
3942 reflections	(Δ/σ)_max_ = 0.001
138 parameters	Δρ_max_ = 0.54 e Å^−^^3^
0 restraints	Δρ_min_ = −0.39 e Å^−^^3^

### Special details

**Table:**

Experimental. The crystal was placed in the cold stream of an Oxford Cyrosystems Cobra open-flow nitrogen cryostat (Cosier & Glazer, 1986) operating at 100.0 (1) K.
Geometry. All e.s.d.'s (except the e.s.d. in the dihedral angle between two l.s. planes) are estimated using the full covariance matrix. The cell e.s.d.'s are taken into account individually in the estimation of e.s.d.'s in distances, angles and torsion angles; correlations between e.s.d.'s in cell parameters are only used when they are defined by crystal symmetry. An approximate (isotropic) treatment of cell e.s.d.'s is used for estimating e.s.d.'s involving l.s. planes.
Refinement. Refinement of *F*^2^ against ALL reflections. The weighted *R*-factor *wR* and goodness of fit *S* are based on *F*^2^, conventional *R*-factors *R* are based on *F*, with *F* set to zero for negative *F*^2^. The threshold expression of *F*^2^ > σ(*F*^2^) is used only for calculating *R*-factors(gt) *etc*. and is not relevant to the choice of reflections for refinement. *R*-factors based on *F*^2^ are statistically about twice as large as those based on *F*, and *R*- factors based on ALL data will be even larger.

### Fractional atomic coordinates and isotropic or equivalent isotropic displacement parameters (Å^2^)

**Table:**

	*x*	*y*	*z*	*U*_iso_*/*U*_eq_	
Br1	0.741696 (15)	1.042853 (7)	0.08809 (2)	0.01677 (4)	
N1	0.78272 (13)	0.79718 (7)	0.17705 (16)	0.01325 (19)	
H1N1	0.712 (2)	0.7551 (11)	0.133 (3)	0.022 (4)*	
N2	0.99471 (14)	0.70859 (7)	0.34946 (18)	0.0152 (2)	
H2N2	1.091 (3)	0.7028 (12)	0.426 (3)	0.031 (5)*	
H1N2	0.926 (2)	0.6673 (12)	0.296 (3)	0.036 (5)*	
C1	0.72188 (15)	0.87250 (8)	0.11482 (19)	0.0142 (2)	
H1A	0.6112	0.8790	0.0301	0.017*	
C2	0.82285 (16)	0.93842 (8)	0.1764 (2)	0.0139 (2)	
C3	0.99045 (15)	0.92827 (8)	0.3065 (2)	0.0148 (2)	
H3A	1.0606	0.9729	0.3486	0.018*	
C4	1.04836 (15)	0.85246 (8)	0.36957 (19)	0.0144 (2)	
H4A	1.1575	0.8455	0.4585	0.017*	
C5	0.94285 (15)	0.78397 (8)	0.30010 (18)	0.0127 (2)	
O1W	0.34331 (12)	0.71054 (7)	0.61811 (17)	0.0206 (2)	
H2W1	0.392 (3)	0.6972 (13)	0.729 (3)	0.038 (6)*	
H1W1	0.396 (3)	0.7487 (15)	0.594 (4)	0.043 (6)*	
O1	0.55652 (11)	0.67725 (5)	0.02333 (15)	0.01630 (18)	
O2	0.75988 (11)	0.58655 (6)	0.15601 (16)	0.01766 (18)	
C6	0.48381 (16)	0.53893 (7)	−0.0202 (2)	0.0139 (2)	
H6AA	0.3763	0.5537	−0.1026	0.017*	
C7	0.61164 (15)	0.60438 (7)	0.06033 (19)	0.0127 (2)	

### Atomic displacement parameters (Å^2^)

**Table:**

	*U*^11^	*U*^22^	*U*^33^	*U*^12^	*U*^13^	*U*^23^
Br1	0.01516 (6)	0.01024 (6)	0.02259 (7)	0.00028 (4)	0.00262 (4)	0.00195 (5)
N1	0.0112 (4)	0.0107 (5)	0.0165 (5)	−0.0015 (4)	0.0023 (4)	−0.0004 (4)
N2	0.0124 (5)	0.0108 (5)	0.0201 (5)	−0.0002 (4)	0.0017 (4)	0.0006 (4)
C1	0.0128 (5)	0.0124 (5)	0.0161 (5)	0.0000 (4)	0.0027 (4)	0.0010 (4)
C2	0.0137 (5)	0.0103 (5)	0.0170 (5)	0.0005 (4)	0.0040 (4)	0.0008 (4)
C3	0.0129 (5)	0.0123 (5)	0.0182 (6)	−0.0025 (4)	0.0035 (4)	−0.0017 (4)
C4	0.0117 (5)	0.0129 (6)	0.0162 (5)	−0.0016 (4)	0.0011 (4)	−0.0019 (4)
C5	0.0111 (5)	0.0128 (5)	0.0139 (5)	−0.0003 (4)	0.0036 (4)	−0.0005 (4)
O1W	0.0163 (5)	0.0189 (5)	0.0221 (5)	−0.0039 (4)	−0.0003 (4)	0.0050 (4)
O1	0.0134 (4)	0.0097 (4)	0.0228 (5)	0.0004 (3)	0.0014 (3)	−0.0001 (3)
O2	0.0120 (4)	0.0126 (4)	0.0245 (5)	0.0001 (3)	0.0002 (3)	−0.0001 (4)
C6	0.0112 (5)	0.0111 (5)	0.0176 (5)	−0.0005 (4)	0.0016 (4)	−0.0011 (4)
C7	0.0130 (5)	0.0105 (5)	0.0138 (5)	−0.0010 (4)	0.0030 (4)	−0.0001 (4)

### Geometric parameters (Å, °)

**Table:**

Br1—C2	1.8832 (13)	C3—H3A	0.9300
N1—C5	1.3556 (15)	C4—C5	1.4223 (17)
N1—C1	1.3616 (16)	C4—H4A	0.9300
N1—H1N1	0.899 (18)	O1W—H2W1	0.77 (2)
N2—C5	1.3278 (16)	O1W—H1W1	0.81 (2)
N2—H2N2	0.81 (2)	O1—C7	1.2863 (15)
N2—H1N2	0.89 (2)	O2—C7	1.2416 (14)
C1—C2	1.3620 (17)	C6—C6^i^	1.326 (2)
C1—H1A	0.9300	C6—C7	1.4990 (17)
C2—C3	1.4130 (17)	C6—H6AA	0.9300
C3—C4	1.3635 (18)		
			
C5—N1—C1	122.64 (11)	C2—C3—H3A	120.3
C5—N1—H1N1	119.8 (11)	C3—C4—C5	120.37 (11)
C1—N1—H1N1	117.5 (12)	C3—C4—H4A	119.8
C5—N2—H2N2	116.7 (14)	C5—C4—H4A	119.8
C5—N2—H1N2	119.8 (13)	N2—C5—N1	119.22 (11)
H2N2—N2—H1N2	123.4 (19)	N2—C5—C4	122.97 (11)
N1—C1—C2	120.10 (11)	N1—C5—C4	117.81 (11)
N1—C1—H1A	119.9	H2W1—O1W—H1W1	105 (2)
C2—C1—H1A	120.0	C6^i^—C6—C7	123.34 (14)
C1—C2—C3	119.67 (12)	C6^i^—C6—H6AA	118.3
C1—C2—Br1	120.62 (9)	C7—C6—H6AA	118.3
C3—C2—Br1	119.71 (9)	O2—C7—O1	124.24 (11)
C4—C3—C2	119.37 (11)	O2—C7—C6	120.03 (11)
C4—C3—H3A	120.3	O1—C7—C6	115.72 (11)
			
C5—N1—C1—C2	−0.12 (18)	C1—N1—C5—N2	−178.04 (12)
N1—C1—C2—C3	−0.51 (19)	C1—N1—C5—C4	1.59 (17)
N1—C1—C2—Br1	178.98 (9)	C3—C4—C5—N2	177.11 (13)
C1—C2—C3—C4	−0.42 (19)	C3—C4—C5—N1	−2.50 (18)
Br1—C2—C3—C4	−179.92 (10)	C6^i^—C6—C7—O2	6.0 (2)
C2—C3—C4—C5	1.93 (19)	C6^i^—C6—C7—O1	−173.89 (16)

Symmetry codes: (i) −*x*+1, −*y*+1, −*z*.

### Hydrogen-bond geometry (Å, °)

**Table:**

*D*—H···*A*	*D*—H	H···*A*	*D*···*A*	*D*—H···*A*
N1—H1N1···O1	0.902 (18)	1.815 (18)	2.7136 (14)	174.1 (19)
N2—H2N2···O1W^ii^	0.82 (2)	2.11 (2)	2.9143 (16)	169.7 (19)
N2—H1N2···O2	0.893 (19)	1.946 (19)	2.8348 (15)	173.2 (19)
O1W—H2W1···O1^iii^	0.77 (2)	2.07 (2)	2.8213 (15)	169 (2)
O1W—H1W1···O1^iv^	0.82 (3)	1.99 (3)	2.7865 (14)	167 (3)
C3—H3A···O2^v^	0.93	2.41	3.3089 (17)	162.

Symmetry codes: (ii) *x*+1, *y*, *z*; (iii) *x*, *y*, *z*+1; (iv) *x*, −*y*+3/2, *z*+1/2; (v) −*x*+2, *y*+1/2, −*z*+1/2.
